# The Sibaté Asbestos-Cement Facility Case Study: Reconstructing the Production Process to Assess Occupational and Environmental Impacts

**DOI:** 10.5334/aogh.5341

**Published:** 2026-11-12

**Authors:** Margarita Giraldo, Juan Pablo Ramos-Bonilla, Laura Bedoya, Cristian Vargas, Benjamin Lysaniuk, María Fernanda Cely-García, Pietro Comba, Francesco Turci, Milena Maule, Corrado Magnani, Andres Raquejo, Benedetto Terracini, Stefano Silvestri

**Affiliations:** 1 Department of Civil and Environmental Engineering, Universidad de Los Andes, Bogotá, Colombia; 2 Cancer Epidemiology, Department of Medical Sciences, University of Turin and A.O.U. Città della Salute e della Scienza di Torino, CPO-Piemonte, Turin, Italy; 3 Collegium Ramazzini, 40010, Bentivoglio, Bologna, Italy; 4 CNRS, UMR 8586 PRODIG, Aubervilliers, France; 5 G. Scansetti’ Interdepartmental Centre for Studies on Asbestos and Other Toxic Particulates, University of Torino, Turin, Italy; 6 Department of Chemistry, University of Turin, Turin, Italy; 7 Department of Translational medicine, University of Eastern Piedmont and CPO - Piemonte at Novara, Novara, Italy; 8 Unit of Cancer Epidemiology, Regional Operating Center of Piemonte (COR Piemonte), University of Torino and CPO-Piemonte, 10126 Turin, Italy (Retired); 9 Department of Translational medicine, Medical Statistics University of Eastern Piedmont and CPO - Piemonte at Novara, Novara, Italy (Collaborator)

**Keywords:** asbestos, asbestos-cement industry, mesothelioma cluster, environmental surveillance, Sibaté Colombia

## Abstract

*Background:* Despite hosting one of the largest asbestos industries in Latin America, Colombia lacked adequate environmental and occupational surveillance, hindering a full understanding of its operations and impacts. The first asbestos-processing facility began operations in the municipality of Sibaté in 1942, where the first cluster of malignant pleural mesothelioma was recently identified. Areas containing friable asbestos waste have also been documented within the urban zone.

*Objectives:* This study aimed to develop and apply a structured approach to (i) reconstruct historical production practices and operational conditions under which workers may have been exposed to asbestos and (ii) identify potential environmental pathways through which asbestos may have been released from the facility into the surrounding environment.

*Methods:* Thirteen former workers were interviewed to reconstruct key production processes, including handling and disposal of raw asbestos and asbestos-containing materials. In parallel, environmental surveillance records from the regional environmental authority for 1980–2019 were reviewed to identify documented environmental management and monitoring practices.

*Results:* Findings identified operational stages and waste-management practices that may have provided potential pathways for asbestos release, including wastewater discharges, sludge disposal, and landfilling in Sibaté and nearby municipalities. Environmental records began only in 1980, leaving the facility’s first 38 years of operation (1942–1979) without documented environmental oversight, and included no asbestos-specific monitoring or quantitative exposure data.

*Conclusions:* The Sibaté case shows how asbestos risks persist beyond industrial boundaries, while this study provides a practical framework for countries facing similar surveillance limitations. Findings highlight the need for transparent data, sustained environmental surveillance, and comprehensive remediation to address the long-term environmental and public health consequences of asbestos-contaminated sites.

## Introduction

Asbestos is a group of naturally occurring mineral fibers extensively used due to their physicochemical properties, particularly in the construction and automotive industries [1]. Since the late 1970s, the International Agency for Research on Cancer (IARC) has classified all forms of asbestos as carcinogenic to humans [2]. Asbestos causes neoplastic diseases, including malignant pleural mesothelioma (MPM), as well as cancers of the lung, larynx, and ovary, and non-neoplastic diseases such as asbestosis [2].

Colombia’s asbestos industry began in 1942 with the establishment of an asbestos-cement plant (ACP) in Sibaté, 25 km from Bogotá [3]. Worldwide, these types of industries have been associated with increased disease rates among both workers and communities near former mining and manufacturing sites, including in Italy, the United States, and Canada [4–6]. After several unsuccessful regulatory attempts, Colombia banned asbestos through Law 1968 of 2019, effective by January 1, 2021; 72 countries now have asbestos bans [7, 8].

In Sibaté, a municipality of 39,817 inhabitants in 2017 (19,817 females and 20,000 males) [9], 15 MPM cases diagnosed between 2008 and 2016 were identified through 355 door-to-door surveys. All diagnoses were validated by a panel of physicians using criteria from the Italian National Mesothelioma Registry (ReNaM) [10]. Notably, only one case was associated with occupational exposure, while the others were linked to para-occupational or environmental exposure. Age-standardized incidence rates were 3.1 per 100,000 person-years for men and 1.6 for women [9], comparable to those observed in other mesothelioma clusters. Sibaté therefore represents the first recognized MPM cluster in Colombia. These findings emerged from a research program initiated in 2015 in response to community concerns, which has produced several peer-reviewed studies through an international collaboration [11, 12].

Colombia’s regional environmental governance framework dates back to 1961, with the establishment of the Autonomous Regional Corporations (CARs) as public entities with administrative and financial autonomy. Since 1993, the Ministry of Environment has been responsible for national environmental policy, while the CARs regulate, monitor, and enforce compliance within their jurisdictions for activities with potential environmental impacts, including air emissions, wastewater discharges, and soil contamination. As such, they serve as the primary authorities for environmental surveillance and control at the regional level [13].

Despite this institutional structure, Colombia’s environmental management system remains decentralized and complex. Although environmental information is formally public, access is often limited in practice due to fragmented information systems and poor data integration. These constraints hinder access to environmental data and reduce the effectiveness of governance.

Risk assessment at former asbestos-cement sites is challenging because environmental, occupational, and epidemiological surveillance has been limited and asbestos-related diseases have long latency periods (e.g., up to 50 years for mesothelioma) [14]. In Sibaté, this limitation is more pronounced: the absence of routine environmental and occupational sampling to quantify asbestos fiber concentrations hinders robust reconstruction of historical exposures among workers and nearby communities. Retrospective exposure assessment (REA) methods, which rely on qualitative information such as expert judgment and self-reported histories, can partially address these uncertainties but cannot replace measurement-based quantitative analysis [15].

In the absence of comprehensive environmental surveillance and enforcement records, this study aims to develop and apply a structured approach to reconstruct historical production practices and operational conditions under which workers may have been exposed to asbestos, and to identify potential environmental pathways through which asbestos may have been released from the facility into the surrounding environment. By integrating former-worker testimonies with environmental surveillance records from the CAR, we identify plausible pathways of asbestos fiber release and assess the strengths and limitations of the available qualitative evidence. The approach provides a framework applicable to other legacy asbestos sites where quantitative historical data are unavailable.

## Methods

### Identification of facility areas with higher potential for asbestos exposures

To identify areas within the Sibaté ACP with higher potential for asbestos exposure, a survey (Supplementary File 1) was administered to former workers. The survey collected information on: (i) Location-specific roles and time spent in the facility; (ii) the production process, from raw material receipt to product dispatch; (iii) the spatial organization of operational areas; (iv) asbestos control systems in place; (v) indoor cleaning procedures; and (vi) waste management practices.

Participants were recruited through prior community-based workshops conducted in 2018 and 2019, and through local community members. They were long-term residents who lived in the municipality during the period when asbestos landfill areas were constructed in Sibaté. No exclusion criteria were applied, and no financial or other incentives were offered. Of 54 individuals contacted, 23 were former workers, 13 of whom agreed to participate (participation rate: 57%). Interviews were conducted remotely between 2020 and 2021 by videoconference (*n* = 11) or telephone (*n* = 2). Collectively, participants’ work histories at the facility spanned from the late 1960s to the early 2010s. All interviews were audio-recorded with informed consent.

During virtual interviews, historical aerial photographs (1957–2009) from the Agustín Codazzi Geographic Institute (IGAC) were used to help participants identify and locate plant operations. Based on the participants’ accounts, production flow diagrams were developed to identify tasks and locations associated with higher potential of asbestos exposure, particularly those involving dry processes and manual handling. Information on asbestos landfill practices and potential disposal sites within and around Sibaté was also collected.

In the absence of quantitative measurements, a qualitative assessment was conducted to describe the potential for asbestos release across similar exposure groups (SEGs) within the ACP. This REA followed established principles of expert judgment, drawing on historical information on production processes, job tasks, and material handling [15, 16]. The reconstruction was based on former-worker testimonies describing production activities, the physical form of asbestos-containing materials (ACMs), and the processes most likely to generate airborne fibers. Information on engineering controls and personal protective equipment (PPE) was available only at the plant level and could not be assigned consistently to individual SEGs.

### Review of environmental surveillance records of the ACP

The ACP environmental surveillance records were obtained through publicly accessible CAR databases: the File Management System (SAE) and the Public Documentary Information Management System (SIDCAR) [17, 18]. Although these platforms provide public access to environmental information, consultation may be restricted from outside the country due to VPN restrictions. Records that could not be accessed and additional information were obtained through formal information requests (right to petition) submitted to CAR and the National Institute of Hydrology, Meteorology, and Environmental Studies of Colombia (IDEAM). Supplementary Material 2 describes the retrieval procedures and lists the CAR records (name and identification code) used in the analysis.

Retrieved documentation included permit files, inspection reports, and compliance records related to environmental management at the ACP. Records were reviewed across key environmental domains, including wastewater discharges, air emissions, and waste management. Only categories relevant to potential asbestos release were included in the analysis.

Timelines were developed to trace practices related to potential asbestos release into air, water, and soil, from the earliest to the most recent records. The analysis examined potential release sources, management practices, treatment systems, and the presence or absence of asbestos-specific monitoring data relevant to potential contamination and exposure pathways. Although the primary aim was to identify quantitative asbestos concentration data, the Results section highlights its absence. Environmental records were qualitatively compared with worker testimonies to assess consistency between reported practices and regulatory records.

## Results

### Identification of facility areas with higher potential for asbestos exposures

Thirteen former ACP workers in Sibaté were interviewed to reconstruct the production process and identify potential asbestos exposure within the facility. Participants worked across multiple operational areas, including raw material handling, production, maintenance, cleaning, and administration, between the 1960s and early 2010s, with employment durations ranging from a few years to more than two decades. Most reported tasks in more than one area, indicating overlapping exposure conditions. Tasks included milling, mixing, shaping, corrugating, demolding, calendering, and finishing. Additional duties included quality control, waste recovery, product dispatch, painting, and general cleaning.

Based on the interviews, production followed the *Hatschek process,* combining dry and wet stages [19]. Workers consistently identified unloading, storage, and milling of raw asbestos as the most dust-intensive tasks. Asbestos fibers were received in bags and manually unloaded from rail wagons (until approximately 1980) or trucks. When mechanical systems were unavailable, workers carried sacks on their shoulders to reach the crane, a task repeatedly described as generating visible dust. Opening bags and dry mixing before slurry preparation were also identified as activities with potential for asbestos fiber release. No detailed information was available regarding either the material composition of the asbestos bags or the disposal practices for the empty sacks.

Both asbestos and cement were then transported by forklifts to the facility milling area, where they were dry-mixed and combined with water to form a slurry. The mixture moved to dewatering and preform formation, where it was sieved and placed onto a continuous moving felt. As the material accumulated on a rotating drum, thin layers gradually formed a continuous sheet (preform) of the desired thickness. The preform was then routed to one of three production lines. In the sheet line, mechanized systems produced corrugated roofing tiles and flat panels. The pipe line manufactured asbestos-cement pipes of various diameters and thicknesses, either for drainage or electrical and telecommunications networks and for water distribution. The molding area relied on manual labor to produce items such as storage tanks, elbows, skylights, and roof accessories (Figure 1). Supplementary File 3 shows flowcharts of the three production processes reconstructed from workers’ interviews, along with a glossary of terms.

**Figure 1:**
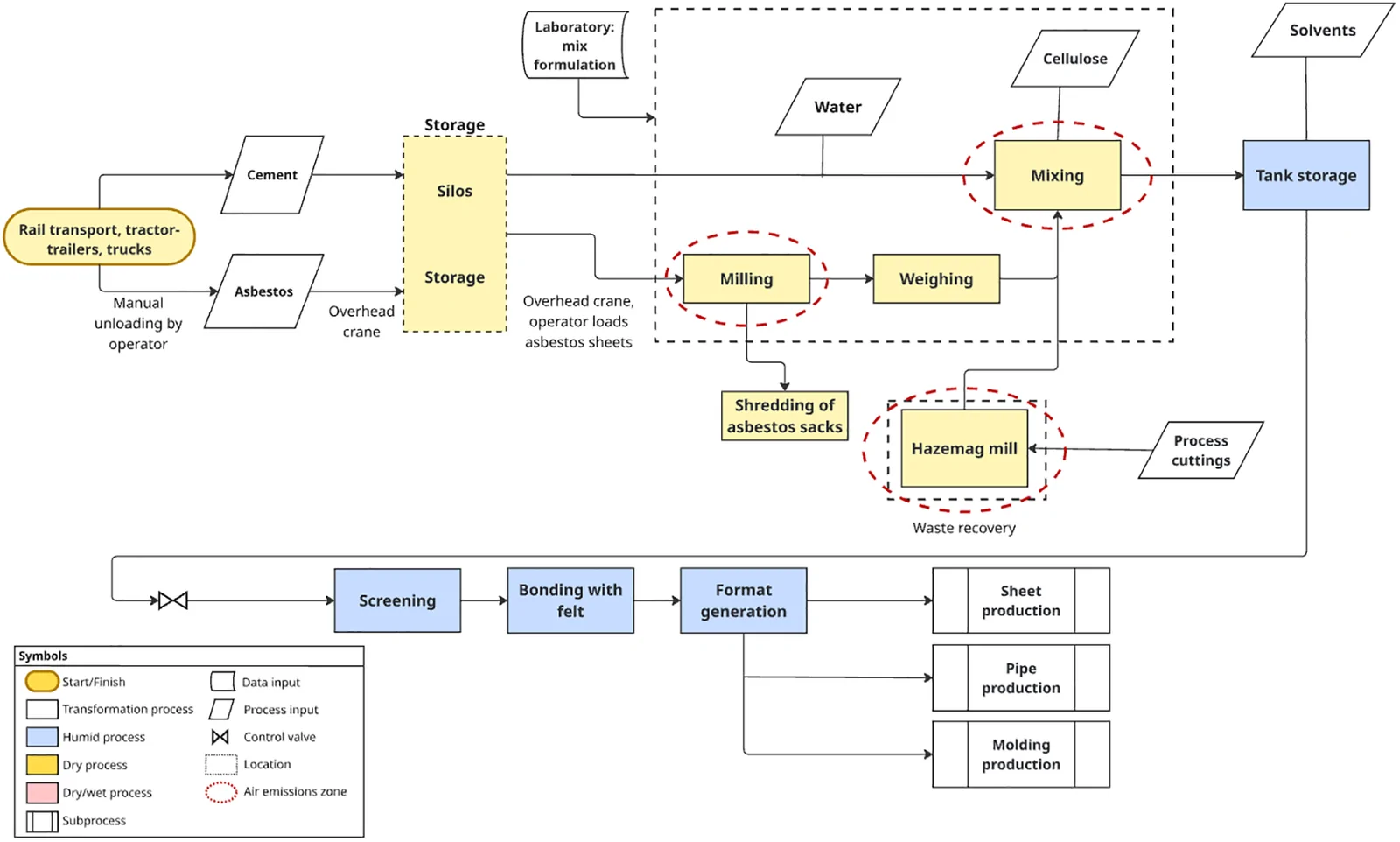
Overall production flowchart from raw material receipt to the initial stages of the three production lines. This figure was developed using information collected from the former workers who participated in the study.

According to former workers, these operational areas were arranged in a linear production sequence across the plant, although the internal partitioning of workshops within individual buildings could not be determined, as has also been reported for other asbestos-cement facilities [20]. Overall, the reconstructed production processes and associated occupational conditions were broadly consistent with those historically described in asbestos-cement manufacturing plants worldwide [17, 19]. Finishing operations in pipe production were reported as areas with higher potential for airborne asbestos dust, while routine cleaning practices, including dry sweeping and the handling of settled dust, may have contributed to fiber resuspension [21]. Similarly, according to worker accounts and known mechanisms of fiber disturbance, routine cleaning practices such as dry sweeping and handling of settled dust had the potential to cause resuspension of asbestos fibers.

Waste management activities were also identified as high-risk. Workers described collecting production residues, including fragments of sheets, pipes, and molded products, which were transported to the Hazemag mill for crushing into fine particulate material in order to be recycled in the process. They emphasized that residues could not be moistened because this reduced grinding efficiency. Consequently, waste handling often involved continuous manipulation of dry, friable material. Similar production and waste-handling practices have been described in other asbestos-cement facilities [22].

Based on worker accounts, the types of asbestos used at the facility appeared to vary over time. Five workers identified asbestos by mineral name, while six did so only by color (blue, white/gray, brown, and yellow fibers). Two did not know which type was used in the production process (Table 1).

**Table 1:** Reported knowledge of asbestos types among former workers.

ID	ASBESTOS USED REPORTED						
	BY TYPE OF MINERAL			BY TYPE OF COLOR			NR
	CHRY	CRO	AM	WHITE/GREY	BLUE	BROWN	
W1							**X**
W2							**X**
W3				○	○		
W4	●	●	●				
W5				○		○	
W6				○			
W7	●				○		
W8	●	●					
W9				○	○		
W10				○	○		
W11	●				○		
W12				○			
W13	●						

Acronyms: Chry—Chrysotile; Cro—Crocidolite; Am—Amosite. ○: Workers referred to asbestos types based on color only (e.g., white, blue, brown), without naming specific types. *NR = X – Workers who reported not knowing the asbestos type.

According to worker testimonies, crocidolite was reportedly used until the early 1980s, when its use was discontinued following growing recognition of its carcinogenic potential at the facility. No information on the period of amosite use was reported. Three of the 13 workers stated that crocidolite was imported from Canada; however, this may reflect recall bias, as Canada never mined or exported crocidolite. One respondent mentioned South Africa as another source of crocidolite. Additionally, three workers reported that chrysotile used at the facility originated from the Campamento mine in Antioquia—the country’s main asbestos mine. Historical records indicate that from 2000 to 2013, the mine produced 4246 and 9296 metric tons annually [23]. After a 2-year closure, operations resumed in 2013 [24], reaching a peak of 13,240 metric tons in 2015 [23].

Grouping the activities by SEGs, raw material handling and waste management may have the highest risk, driven by direct handling of dry asbestos. Overall, 77% of workers reported involvement in more than one SEG, suggesting cumulative and overlapping exposures within the facility. Detailed descriptions for all SEGs are presented in Table 2.

**Table 2:** Summary of worker-reported production steps and associated exposure risks. The table was developed by the authors based on testimonies provided by the former 13 workers who participated in this study.

SEG	KEY TASKS	MATERIAL FORM	POTENTIAL FOR ASBESTOS FIBER RELEASE
Handling raw asbestos	Unloading raw material	Dry asbestos in bags	Possible direct fiber release during asbestos arrival.
Waste management	Waste recovery, dry cleaning	Dust with asbestos	Resuspension of settled asbestos fibers.
Handling products	Finishing process (i.e., cutting and turning)	Dry product	Fiber release during dry manipulation.
Manufacturing	Mixing and slurry preparation	Dry asbestos in bags and wet mix containing asbestos	Fiber release during bag opening and dry mixing.
	Pipeline production	Combined (wet/dry)	Mainly wet, but potential for splashing or dried residue.
	Molding production	Combined (wet/dry)	
	Sheet production	Combined (wet/dry)	
	Slurry dewatering and preform formation	Wet mix containing asbestos	Wet process, moderate disturbance but potential for splashing or dried residue.
Transportation	Overhead crane operation, forklift operation	Dry asbestos in bags and dry product	Asbestos is contained as a non-friable material in the final product or handling dried materials that may release fibers.
Other	Task not related to the production process	No contact with asbestos	No contact with asbestos.
**Workers in >1 SEG: 10 (77%)**			

Information on engineering controls (i.e., dust control) and PPE was available only at the plant-wide level and could not be linked to specific SEGs. Although local exhaust systems were reportedly introduced between the late 1970s and early 1980s, workers described ventilation as largely dependent on natural airflow (open windows), and information on PPE use was limited.

### Review of environmental surveillance records of the ACP

A review of the two publicly accessible digital platforms managed by the CAR, SAE and SIDCAR, identified 10 permit-related and 3 sanction-related case files associated with the ACP, totaling more than 10,000 pages. These databases were last accessed in September 2025. Details on the reviewed records and information extraction process are provided in Supplementary Material 2.

#### Wastewater management and discharges

The ACP operated for more than seven decades, with available records documenting wastewater management and discharge practices from 1980 to 2017, spanning approximately 37 years. No records concerning wastewater management were identified for the period before 1980. Given that the facility began operations in 1942, this represents a 38-year period for which no environmental oversight by the environmental authority could be documented in the reviewed records. The available documentation describes long-term discharges of industrial and domestic wastewater (IWW/DWW) into the El Muña Reservoir and nearby water bodies [25].

Discharges to El Muña Reservoir were first documented in 1980 and 1985 [25]. Recorded coordinates located the IWW pipeline south of the ACP (Figure 2). In 1989, CAR identified additional discharge points releasing untreated IWW into a drainage channel connected to the Bogotá River and ordered their cessation [25]. Although a wastewater treatment and recirculation system was proposed in 1981, records indicate that untreated discharges persisted until the late 1990s [25].

**Figure 2:**
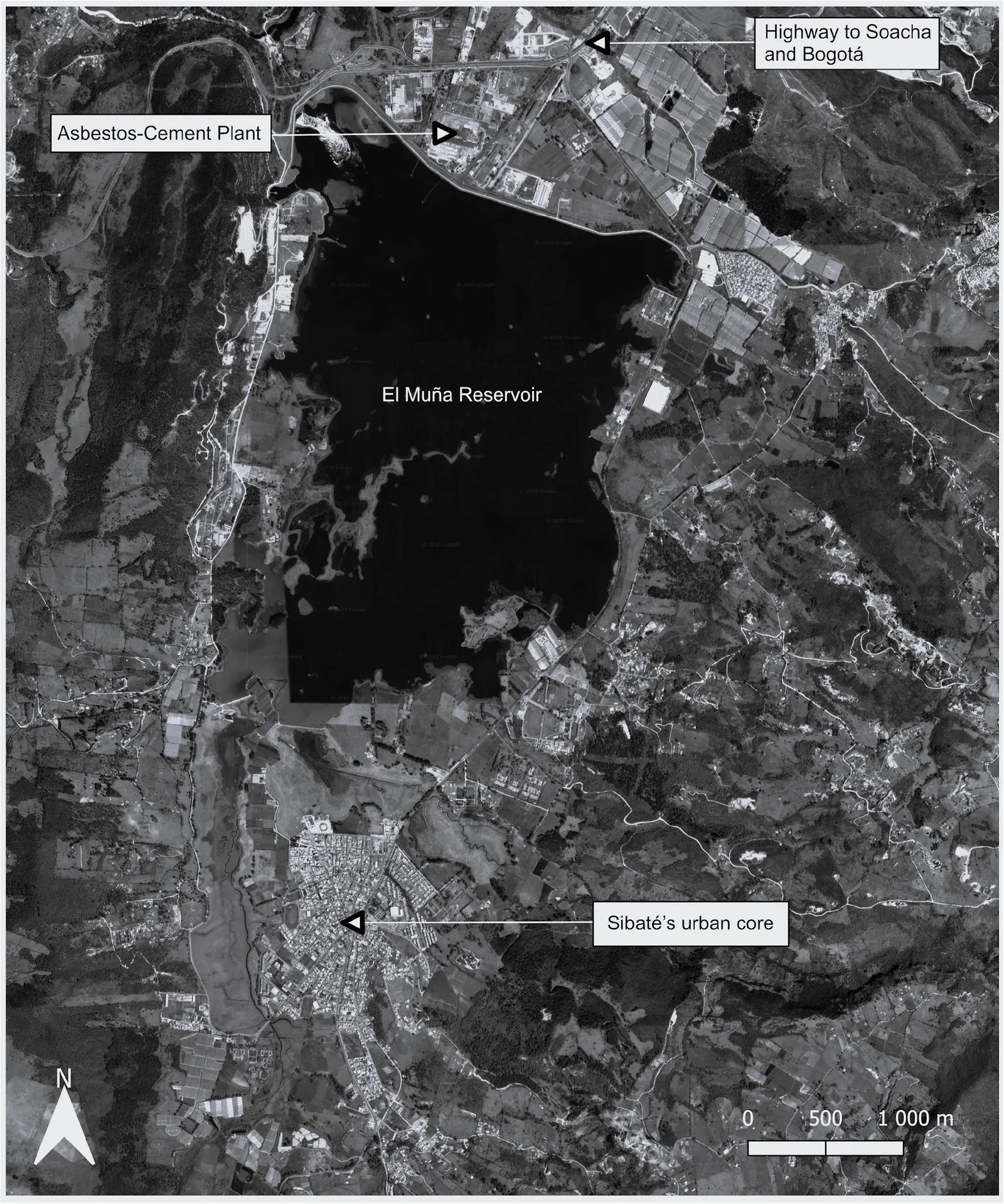
Location of the asbestos cement facility and the El Muña Reservoir. The approximate location of the ACP pipeline was at the south of the ACP, toward the El Muna Reservoir according to information from CAR file No. 31848 [25], accessed through the SAE and SIDCAR platforms.

CAR approved the Industrial Wastewater Treatment Plant (IWWTP) and authorized its operation in late 1988 [25]. However, in 1989, the ACP reported operational failures, including tank overflows and clogged pipelines, which compromised treatment and allowed continued discharges into the reservoir [25]. In 1993, the company reported recovering 85–90% of IWW sludge, with the remainder disposed of in Soacha landfills [25]. Discharges into El Muña Reservoir reportedly ceased only in 1999—11 years after IWWTP approval [25]. Nevertheless, intermittent releases persisted, with the ACP reporting emergency discharges as late as 2011 [25]. By 2017, inspections reported no active discharges [25].

A chronological timeline of environmental events and industrial activities related to potentially asbestos-contaminated discharges is presented in Figure 3.

**Figure 3:**
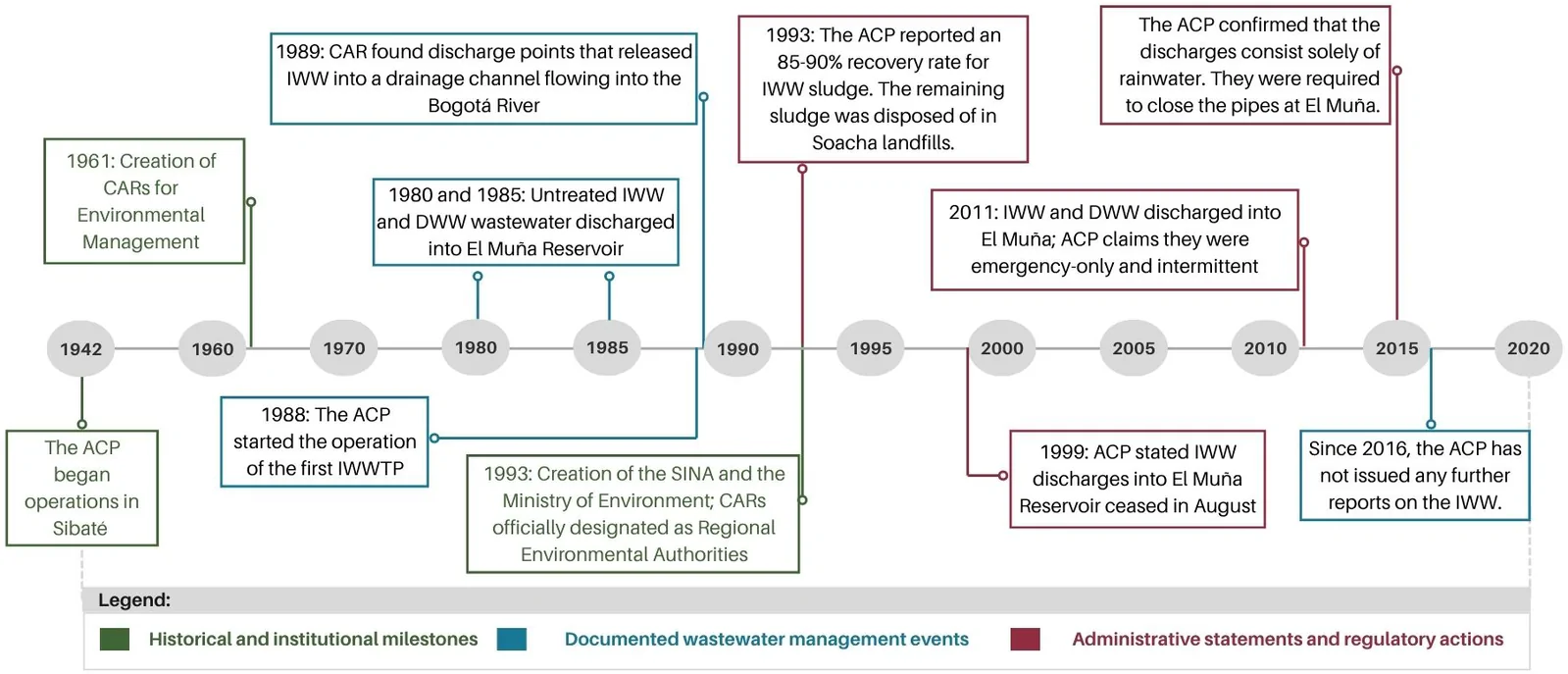
Timeline of industrial wastewater management at the ACP. Acronyms: ACP—Asbestos Cement Plant; CAR—Regional Autonomous Corporation of Cundinamarca; SINA—National Environmental System; IWW—Industrial Wastewater; IWWTP—Industrial Wastewater Treatment Plant; DWW—Domestic Wastewater. This timeline was developed using the information reported in the Environmental Authority administrative files (CAR file No. 31848) [25].

Although the review aimed to identify asbestos concentration measurements in industrial effluents, none were found in available records [25]. Documentation included only basic regulatory laboratory analyses, with no asbestos-specific parameters or hazardous classification of sludge or wastewater. In 2007, CAR required a Toxicity Characteristic Leaching Procedure (TCLP) test to assess sludge toxicity. However, no documentation confirmed that it was conducted [25]. By 2009, the company reported recirculating all sludge into production and stated that TCLP analysis was unnecessary [25]. Although the sludge may have contained asbestos due to its origin within the asbestos-cement production process, no official classification of this sludge as hazardous waste was identified [25]. Consequently, the records do not allow estimation of discharged asbestos quantities, though these undocumented discharges represent a plausible pathway for community exposure in Sibaté and surrounding areas.

#### Waste management

Environmental authority records on waste management were available for the period 1982–2019. The review identified waste-management practices that may have provided potential pathways for the redistribution of ACMs within Sibaté and surrounding areas. Early documentation provides limited detail, but later records describe disposal of production residues—including sludge and solid waste—at multiple sites.

As with records on industrial wastewater discharge, the earliest digitized record on industrial waste management dates to 1982 [25]. This document does not specify residue-handling procedures but indicates that CAR provisionally accepted existing practices and required the company to prepare a management plan for both wastewater and sludge [25].

CAR documentation also mentions the possible use of solid industrial waste to fill flood-prone or eroded areas. File No. 31848 states: “Initially, flood-prone or eroded lands may be filled, subject to prior study and approval of the project by the Environmental Engineering Division of the Corporation” [25]. However, available records do not clarify whether such studies were conducted or the project was approved [25].

Documentation suggests that waste management practices involved multiple locations over time. Between 1993 and 1994, the ACP reported sending domestic waste to the Mondoñedo and Doña Juana municipal landfills, while some production sludge was reportedly sent to unspecified sites in Soacha [25]. CAR considered this disposal inadequate, instructed the company to stop these practices, and recommended construction of a hazardous-waste landfill [25]. According to the case files, the landfill was never built because the company stated that international discussions considered ACM non-hazardous and that sludge was increasingly recirculated into production [25]. However, the reviewed records provided no scientific references or technical evidence supporting these assertions.

Additional potential disposal sites are described in later records. By 1995, one document indicated that waste might have been disposed of behind the ACP around 1965, while the La Macarena sector of Sibaté was also noted as a possible disposal site [25]. In 2019, a CAR technical inspection documented fill material contaminated with asbestos—including chrysotile, crocidolite, and amosite—at an average depth of 1.1 meters on land adjacent to the ACP [25]. These observations are consistent with findings from our research group, which detected asbestos fibers (chrysotile and crocidolite) in soil samples collected from several public sites in Sibaté, with concentrations ranging from 2% to 10% at depths of 0.1–1.1 meters [9]. While these results demonstrate the presence of asbestos in local soil, the available evidence does not allow attribution to a specific source. Figure 4 presents a chronological timeline of documented events and regulatory records related to ACP waste management.

**Figure 4:**
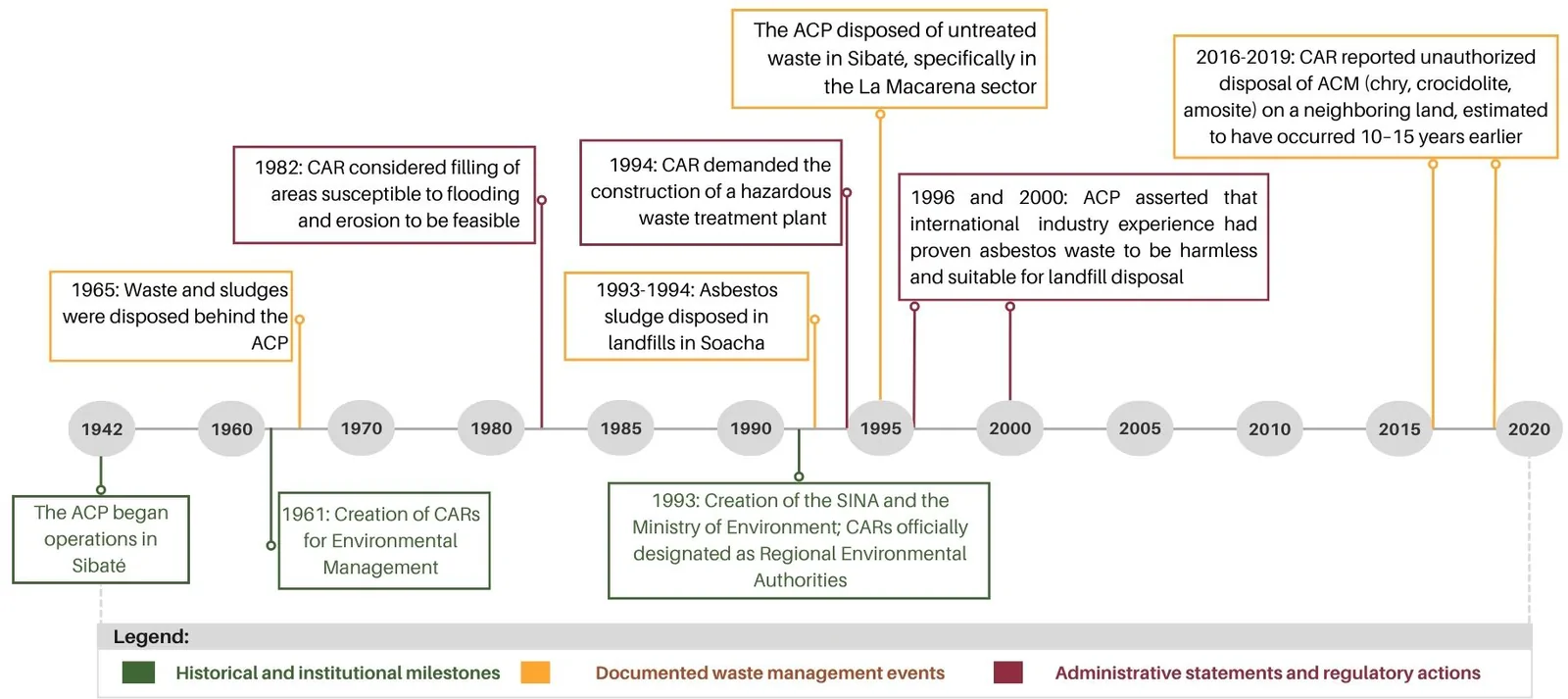
Timeline of industrial solid and sludge waste management at the ACP. Acronyms: ACP—Asbestos Cement Plant; CAR—Regional Autonomous Corporation of Cundinamarca; SINA—National Environmental System; ACM – Asbestos containing materials; Chry—Chrysotile. This timeline was developed using the information reported in the Environmental Authority administrative files (CAR files No. 74817 and 31848) [25].

As with industrial wastewater discharges, no quantitative records describe the total volume or concentration of asbestos released as industrial waste over the facility operating history [25]. Limited registry data documented annual quantities of asbestos waste sent for disposal ranging from 232 to 6900 kg across 6 reporting years (2008–2023; Supplementary File 4) [26].

Over the subsequent three decades, multiple entries in environmental case files document waste handling and disposal activities that may represent potential pathways for the redistribution of ACMs in areas near the facility [25]. Worker testimonies complement these records by describing the transport of asbestos-containing waste, such as broken pipes, sheets, and fragments, for use as construction fill, sometimes upon request of residents. These accounts indicate reported redistribution of waste materials beyond the facility (i.e., with reported frequencies of up to five truckloads per week), which may have created potential pathways for community exposure in Sibaté and Soacha. Workers also reported that asbestos-containing waste was disposed of at El Muña Reservoir and nearby neighborhoods, consistent with descriptions in a case file from 1982 [25]. According to participants, these practices were “common from the early years of the plant until the early 1980s, when the first cases of asbestosis began to be known in the region.*”* It is unclear which specific cases of asbestosis were being recognized during this period, as the available information is limited to workers’ statements.

#### Air emissions

Available records on air emissions began in 1990, leaving a substantial gap for earlier decades. Monitoring activities primarily addressed pollutants regulated under national standards and did not systematically include asbestos. The reviewed records contained information from 1990 to 2017, mostly related to the management of atmospheric emissions from stationary sources in compliance with Colombia’s Resolution 909 of 2008 [27]. This regulation requires periodic monitoring of pollutants generated by fuel combustion processes, including particulate matter, sulfur dioxide, and nitrogen oxides, but does not establish asbestos-specific monitoring requirements [25].

Environmental compliance records from 2005 to 2009 include brief reports of chrysotile concentrations at selected fixed locations within the facility, including the bag opener, scrap milling area, central dust control system, and high-vacuum plate system [25]. These were stationary source-emission and area measurements, taken at fixed points near equipment rather than as personal samples of workers’ breathing-zone exposure, consistent with the ISO 10397:1993 stationary-source emission method they reference [28, 29], and with the European Council Directive 87/217/EEC for determining asbestos emissions from stationary sources [30]. The reports indicate only that concentrations were below 0.1 f/cc. They, therefore, do not constitute quantitative occupational exposure measurements, and the available documentation did not include sufficient methodological information, such as sampling duration, equipment, sampling strategy, analytical details, or the exact sampling locations, to reconstruct historical occupational exposure levels. On this basis, these data were considered insufficient, on their own, to reconstruct historical occupational exposure or to reliably quantify asbestos emissions from the facility.

Notably, no asbestos-specific emission standards existed in Colombia during this period. By 2017, company records reported that asbestos had been fully replaced with alternative raw materials across all ACP production lines [25].

## Discussion

This study integrates worker testimonies with environmental regulatory records to reconstruct historical occupational practices and potential environmental releases from an ACP in Sibaté, Colombia. In the absence of systematic quantitative monitoring, this qualitative approach provides a structured framework for identifying critical exposure processes, pathways, and institutional gaps.

Both testimonies and regulatory records document long-term wastewater discharges and disposal of sludge and industrial waste. Although asbestos concentrations were not measured in these materials, the documented practices indicate potential pathways through which ACMs may have been released or redistributed beyond the facility. The triangulation of information from both sources is presented in Supplementary Material 5.

Testimonies of former workers identified the highest potential fiber release during milling, raw material reception, mixing, recovery of cut products, pipe finishing, and waste crushing in the Hazemag mill. These activities were largely performed under dry conditions, with ventilation relying mainly on natural airflow until local exhaust systems were introduced between the 1970s and 1980s, consistent with high-exposure scenarios reported in other plants [31]. Although some improvements were reported, including changing rooms in the 1990s, workers indicated that poor conditions persisted and awareness of asbestos-related risks emerged only in the late 1980s–1990s, despite well-established international evidence of carcinogenicity [1, 2].

Because no quantitative occupational exposure measurements are available for the Sibaté ACP, airborne fiber concentrations reported for asbestos-cement facilities in other countries provide contextual information only. One study reported average airborne fiber concentrations in the Dutch asbestos-cement industry ranging from 7.46 f/ml (1970–1974) to 0.22 f/ml (1985–1989) during raw material handling, and from 6.16 to 0.21 f/ml during finishing of dry products [32]. These reductions coincided with the introduction of local exhaust ventilation systems similar to those reported in this study. Although these findings cannot be directly extrapolated to Colombia, they provide contextual evidence that comparable asbestos-cement tasks have historically been associated with substantial airborne fiber concentrations, particularly before the implementation of effective dust controls [32].

Estimating national asbestos use in Colombia is challenging because of limited official records. However, *Legiscomex* trade data indicate that between 2009 and 2019, the Sibaté ACP imported an average of 2274 tons of raw asbestos annually, peaking at 5955 tons in 2012 [33]. Until 2015, these imports represented approximately 23% of total national imports. After a sharp decline in 2016 (160 tons), imports rose again to 2560 tons in 2017 (62% of the national total). No imports were recorded from 2018 onward [33].

Environmental oversight has historically been limited. The plant operated for nearly two decades before the establishment of the CARs in 1961 [13], while subsequent regulation and monitoring remained limited. Access to facility-specific hazardous waste data also remains restricted, as national reporting systems aggregate information at municipal or regional levels, limiting public access to facility-specific data. Although environmental information is formally public, difficulties in accessing complete records may limit the effective implementation of the “right to know” principle [34].

International comparisons highlight the long-term persistence and remediation challenges associated with asbestos contamination at historically affected sites. In Casale Monferrato (Italy), remediation efforts have been ongoing for more than three decades [35]. Documented pathways of asbestos dispersion—including transport in uncovered vehicles, redistribution of waste, and informal community use of ACM [35]—show parallels with practices reported in Sibaté [25]. Similar challenges have been documented in other asbestos-affected communities such as Libby (United States), where remediation has required substantial, long-term intervention [36, 37].

In the Sibaté area, nearly one million residents are estimated to live within 10 km of the ACP [38], underscoring the public health importance of identifying whether historical exposure pathways remain active in the surrounding community. Although Colombia’s asbestos ban represents a major milestone, gaps remain in a hazardous waste management plan, long-term follow-up of populations with potential exposure, and national remediation strategies [7]. Addressing these challenges will require identifying ongoing exposure pathways, implementing mitigation measures, and prioritizing cleanup in heavily impacted areas through both national political commitment and international cooperation [39].

Additional exposure pathways may have extended beyond occupational settings. Evidence from international studies indicates that sacks used for raw asbestos transport may constitute a secondary source of exposure when reused in domestic or agricultural contexts [40, 41]. Although the material composition of sacks used in Sibaté could not be confirmed from worker testimonies, previous population-based interviews indicated that some Sibaté residents had access to used sacks in which raw asbestos had been transported [9]. According to these accounts, the sacks were reused for various purposes, including floor coverings inside homes. This practice suggests a potential secondary pathway of para-occupational or community exposure, although exposure itself was not assessed in this study.

Similarly, pipe manufacturing may represent a task with high potential for asbestos fiber release based on evidence from other asbestos-cement facilities [42]. High-pressure pipe production typically uses long chrysotile fibers and, in some cases, crocidolite (blue asbestos) at proportions of up to 20%, generating substantial finishing waste [42]. In Casale Monferrato, pipe waste (“polverino”) was distributed free to the inhabitants and reused in construction. Although some Sibaté workers reported the use of crocidolite, available information was insufficient to confirm whether high-pressure pipes were produced. Further investigation is needed to determine whether these practices represented relevant exposure pathways in Sibaté.

Methodological limitations should be considered. First, the reconstruction relies on retrospective worker testimonies, which are subject to recall bias and may not capture temporal variability in plant operations. Second, participation was voluntary, and recruitment was conducted through community networks; therefore, selection bias cannot be excluded, and participants may have had greater knowledge of asbestos-related issues. Consequently, the representativeness of the interviewed workers cannot be formally assessed. Finally, the absence of quantitative measurements limits the ability to estimate historical exposure levels. Nevertheless, participants represented multiple operational areas and employment periods, and the consistency between independent data sources, worker accounts, and regulatory records strengthens the credibility of the reconstruction.

Overall, this study underscores the importance of combining multiple qualitative data sources to address critical knowledge gaps at historically contaminated sites. This approach can provide valuable information for identifying potential exposure pathways, informing risk assessment, and guiding future environmental and public health interventions.

## Conclusion

The history of the Sibaté ACP shows how asbestos-related risks can extend beyond facility boundaries and persist over time in surrounding communities, particularly in settings where regulatory oversight has been limited. The findings highlight the importance of ensuring transparent access to environmental information, conducting further environmental investigation to determine whether historical release pathways remain active, supporting the long-term follow-up of populations with potential asbestos exposure, and implementing remediation where contamination is confirmed. Although Colombia’s asbestos ban represents an important turning point, sustained political commitment and international cooperation will be essential to addressing the long-term impacts of asbestos exposure on affected populations.

## Data Availability

The data supporting this study’s findings were sourced from two publicly accessible digital platforms maintained by the regional environmental authority (CAR): the File Management System (SAE, for its Spanish acronym; https://sae.car.gov.co/modulos/saepublico/Busqueda.aspx), and the Public Documentary Information Management System (SIDCAR, for its Spanish acronym; https://sidcar.car.gov.co/Modulos/Buzon/ConsultaPublica.aspx). The information is public and can also be requested by a right-to-know petition to the same regional environmental authority.

## Highlights

- Worker testimonies and surveillance records reconstruct past asbestos exposures in Sibaté.
- Plant practices likely dispersed asbestos via wastewater, sludge, and landfills.
- No asbestos-specific environmental surveillance was available for the plant’s first 38 years.
- Risks extend beyond the facility, affecting nearby communities.
- High-risk tasks and areas were identified in the Sibaté plant.

## Glossary

- ACM: Asbestos-containing materials
- ACP: Asbestos cement plant
- Am: Amosite
- ARD: Asbestos-related diseases
- CAR: Regional environmental authority
- Chry: Chrysotile
- Cro: Crocidolite
- DWW: Domestic wastewater
- IARC: International Agency for Research on Cancer
- IDEAM: National Institute of Hydrology, Meteorology, and Environmental Studies of Colombia
- IGAC: Agustín Codazzi Geographic Institute
- IWW: Industrial wastewater
- IWWTP: Industrial Wastewater Treatment Plant
- MPM: Malignant Pleural Mesothelioma
- PPE: personal protective equipment
- REA: Retrospective Exposure Assessment
- ReNaM: National Mesothelioma Registry from Italy
- SAE: File Management System
- SEGs: Similar Exposure Groups
- SIDCAR: Public Documentary Information Management System
- SINA: National Environmental System
- TCLP: Toxicity Characteristic Leaching Procedure
- VPN: Virtual private network

## Additional File

The additional file for this article can be found as follows:

Supplementary material. Detrended Oscillation and Clock Parameters.
