# Supplementary material for: The Sibaté Asbestos-Cement Facility Case Study: Reconstructing the Production Process to Assess Occupational and Environmental Impacts
Source: Ann Glob Health. 2026 Nov 12;92(1):113. doi: 10.5334/aogh.5341 (PMC13761933; doi:10.5334/aogh.5341)
Supplement: Supplementary file 1 [file agh-92-1-5341-s1.pdf]

Supplementary Materials 1: Survey – Questionnaire for Reconstructing the Production Process of the Asbestos-Cement Processing Plant Located in the Municipality of Sibaté, Colombia:

Cuestionario Para el Entendimiento del Proceso Productivo de la Planta de Procesamiento de Asbesto cemento localizada en el Municipio de Sibaté-Colombia

La Universidad de los Andes se encuentra desarrollando un estudio de investigación que tiene como finalidad mejorar nuestro entendimiento acerca de la potencial exposición al asbesto y posibles efectos adversos en la salud de la población del municipio de Sibaté (Colombia).

Actualmente, estamos tratando de determinar si podrían haber ocurrido eventos de dispersión de fibras de asbesto en el aire en la región, y para esto es necesario entender la operación de la planta de asbesto-cemento localizada en el municipio. Es importante caracterizar en el mayor detalle posible el proceso productivo, incluyendo todas las actividades asociadas con el uso, almacenamiento y transformación de las materias primas, y la generación y disposición de residuos.

Lo invitamos a participar en este estudio contestando el cuestionario.

Nombre Del Entrevistador:	Fecha:
Nombre del Entrevistado:	Cédula:
Lugar de Nacimiento:	Fecha de Nacimiento:
Hora de la entrevista:	Dirección del lugar de residencia del entrevistado:
Barrio:	Localidad:
Genero	
Masculino: __	Femenino: ___
Teléfono del entrevistado:	Ocupación actual del entrevistado:
1. ¿Trabajó usted en la planta de asbesto-cemento localizada en el municipio de Sibaté, Cundinamarca?	Sí: ____<br>No: ___

2. ¿Usted vivía en la planta de asbesto-cemento del municipio de Sibaté?

	**Fechas (Ejemplo: 2002 a 2005)**
Sí:	
No:	

3. Indique qué cargos desempeñó, en qué años y el área dentro de la empresa a la cual perteneció:

Cargo	Años	Área	Observaciones

4. Describa detalladamente las actividades que usted desarrolló en la empresa:

____
____

____
____

5. ¿Qué productos se fabricaban en la planta de procesamiento de asbesto durante el periodo que laboró en la planta? ¿Recuerda qué tipo de asbesto se usaba? ¿Recuerda desde que año y hasta cuándo se manufacturó este producto? De un rendimiento de producción aproximada para cada producto.

**Producto (Ejemplo: tubería de asbesto cemento)**	**Tipo de asbesto usado en el proceso (Ejemplo: crocidolita)**	**Años de producción (Ejemplo: 1972 a 1986)**	**Rendimiento de producción aproximado (Ejemplo: 100 tejas por semana)**	**Materias primas asociadas a cada línea de producción (Ejemplo: asbesto)**

Observaciones:

____
____

____
____

Haga una descripción del proceso productivo. Indique en su descripción como llegaba el material, en qué forma llegaba (ejemplo: bultos), cada cuanto llegaba, donde se almacenaba, ¿cuáles procesos generaban más polvo?:

<table>
<tr><td rowspan="4">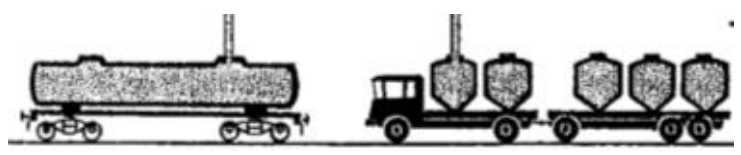</td><td colspan="2">¿Cómo llegaba el material de asbesto a la planta?</td><td></td></tr>
<tr><td colspan="2">¿En qué forma llegaba? (ejemplo: bultos)</td><td></td></tr>
<tr><td colspan="2">¿Cada cuánto llegaba?</td><td></td></tr>
<tr><td colspan="2">¿Dónde se almacenaba?</td><td></td></tr>
<tr><td>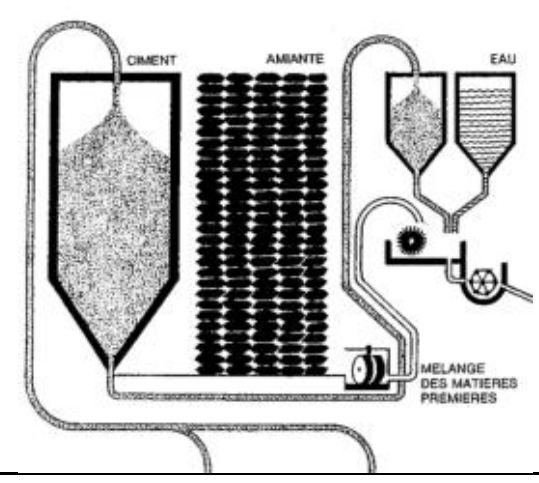
</td><td colspan="2">Después del almacenamiento, ¿a dónde se llevaba el material?</td><td></td></tr>
<tr><td rowspan="16">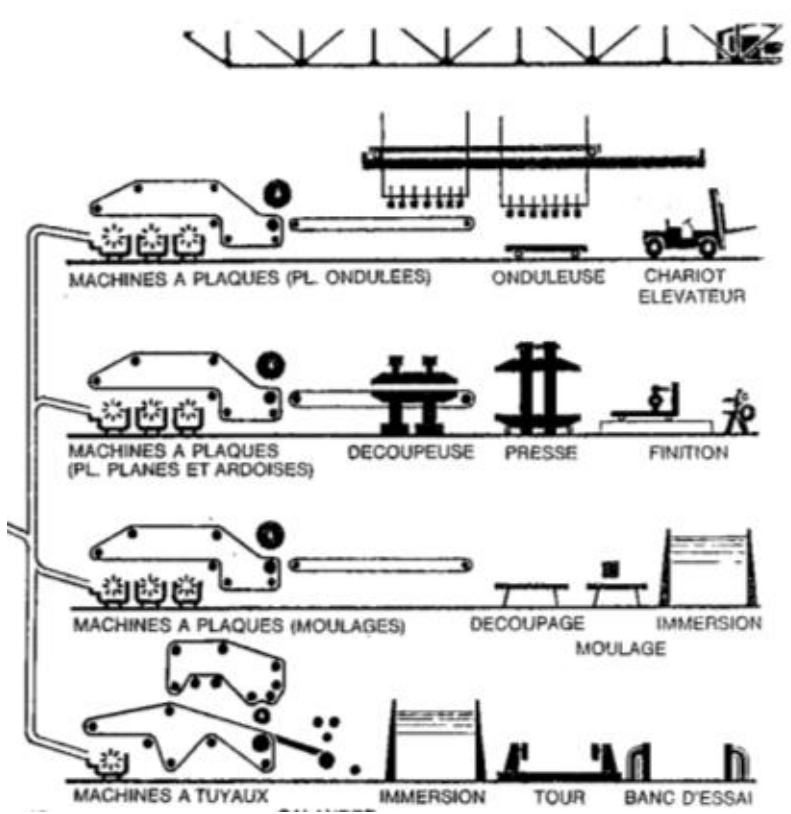
</td><td colspan="3">¿Cómo se llaman los procesos? Para cada línea de producción</td></tr>
<tr><td>Línea 1: Tejas onduladas</td><td colspan="2"></td></tr>
<tr><td>Línea 2</td><td colspan="2"></td></tr>
<tr><td>Línea 3</td><td colspan="2"></td></tr>
<tr><td>Línea 4</td><td colspan="2"></td></tr>
<tr><td>Línea 5</td><td colspan="2"></td></tr>
<tr><td colspan="3">¿Cómo se llaman las máquinas utilizadas en cada uno de los procesos que citó anteriormente?</td></tr>
<tr><td>Proceso</td><td colspan="2"></td></tr>
<tr><td></td><td colspan="2"></td></tr>
<tr><td></td><td colspan="2"></td></tr>
<tr><td></td><td colspan="2"></td></tr>
<tr><td></td><td colspan="2"></td></tr>
<tr><td></td><td colspan="2"></td></tr>
<tr><td></td><td colspan="2"></td></tr>
<tr><td></td><td colspan="2"></td></tr>
<tr><td colspan="2">¿Qué líneas de producción generaban más polvo de asbesto?</td><td>.</td></tr>
<tr><td>¿Considera que las líneas de producción eran continuas o intermitentes? especifique</td><td colspan="3"></td></tr>
</table>

Describa las líneas de producción citadas anteriormente, especifique si los procesos eran secos o húmedos:

____

____

6. Si utilizaba máquinas o herramientas para manipular o trabajar con el asbesto y/o productos que contienen asbesto, haga una descripción de la máquina, del uso de la máquina y su propósito, indique si la frecuencia era continua o intermitente, si era intermitente indique en que frecuencia:

____

____

7. A continuación, se le presentará una fotografía aérea de la planta de asbesto cemento de Sibaté. Podría por favor describir en qué lugares de la planta se llevaban a cabo las actividades productivas que usted describió en la pregunta 5 y 6, y responda las siguientes preguntas:
   - Indique en qué lugar se almacenaba el asbesto.
   - Indique en qué zonas había mayor presencia de polvo de asbesto y asócielas con las líneas de producción.
   - Indique dónde era su puesto de trabajo.
   - En el tiempo que trabajó en la fábrica había fuentes de emisión puntuales de asbesto, como, por ejemplo, ¿ductos o chimeneas que liberaran asbesto al aire exterior? ¿dónde se ubican o ubicaban estos ductos según la fotografía?

____

____

8. Dentro de sus conocimientos por favor contextualice el propósito que tienen los tanques en el lugar donde le especifica el entrevistador en la fotografía aérea, acorde con el proceso productivo descrito anteriormente:

____

____

9. Desarrolle una descripción del interior de la planta en cuanto al tipo de ventanas y su propósito, ¿estás eran cerradas? ¿se utilizaban para iluminación únicamente o para ventilación? ¿por dónde puede entrar y salir el aire al interior de la planta? ¿Las principales entradas y salidas de aire se dan por las compuertas grandes de entrada al edificio?

____

10. Si conoce el sistema de ventilación y aire acondicionado de la **empresa**, haga una descripción a continuación:

11. Describa si existían sistemas de control de contaminación de asbesto en la planta, por ejemplo, sistemas de aspiración en las máquinas, aspersores, controles de humedad que evitaran la liberación de polvo, ciclones, filtros de mangas, precipitador electrostático, confinamiento de los materiales para evitar la acción del viento ¿En qué años comenzaron a ser implementados o siempre existieron?

Tenga en cuenta las siguientes definiciones:

Sistemas de aspiración o aspiradores industriales: dispositivo que succiona aire con el propósito de colectar material de polvo o viruta que permiten un aire más limpio en los lugares de trabajo.

Aspersores industriales: dispositivo que esparce agua en gotas muy finas tipo rocío para evitar la liberación de polvo al aire.

Controles de humedad: cualquier tipo de técnica que humedezca el ambiente de un lugar para evitar la liberación y circulación de partículas finas en un recinto o lugares determinados.

Ciclones: equipo en forma de ciclón que se utiliza para separar partículas suspendidas en el aire de forma que pueda liberar el aire al exterior, pero sin la presencia de polvo o partículas.

Filtros de mangas: dispositivo para la separación de partículas sólidas (polvo) en suspensión de una corriente de aire que tiene la característica de utilizar hileras de mangas de tela en formas de tubos o mangas donde quedan atrapadas las partículas de polvo de la corriente de aire.

Precipitador electrostático: precipitadores electrostáticos son dispositivos que se utilizan para atrapar partículas suspendidas en el aire mediante cargas electrostáticas.

Por favor, contextualice con la fotografía aérea de la planta:

____
____
____
____
____
____

Haga una descripción general del sistema de aseo que se desarrollaba en la empresa, explique ¿quién hacía estas actividades? ¿en este tipo de actividades se liberaba polvo de asbesto? e indique con qué frecuencia se desarrollaban estas actividades:

____
____
____
____

12. Acerca de los residuos sólidos y líquidos de asbesto, responda:

¿Cómo eran los residuos de asbesto? ¿eran polvo, lodos, virutas?	
¿En qué línea de producción se generaban más residuos?	
¿Quién sacaba los residuos de la planta?	
¿Con qué frecuencia y cómo se disponían los residuos sólidos y líquidos de asbesto?	
¿Sabe cómo se disponen actualmente los residuos de asbesto de la planta?	
¿En dónde se almacenaban los residuos? Indique si era al interior o exterior de la planta	Fechas (Ejemplo: 1986 a 1989)
	-

En la fotografía aérea indique en qué lugar de la planta se almacenaban los residuos.

Observaciones:

____
____

13. Responda si tiene conocimiento: ¿Por qué se empezaron a utilizar los residuos de asbesto como rellenos alrededor de la planta y en el Municipio de Sibaté? ¿La acumulación de residuos era mucha que no les cupo y comenzaron a ser utilizados en los rellenos? O ¿les resultaba más económico utilizar el asbesto como relleno que enviarlos a disposición final? ¿Por cuánto tiempo se utilizaron los residuos de asbesto como rellenos?

14. Si conoce acerca de los controles de calidad de los productos, por favor haga una descripción:

____
____
____
____

15. Durante el tiempo que laboró en la empresa existía Administradora de Riesgos Profesionales (ARP), si existía, ¿cuál era el nombre de la (ARP)? Sí no existía, cómo se manejaban los riesgos profesionales.

____
____
____
____

16. ¿Tiene fotos, videos, esquemas u otro tipo de información del interior de la planta que nos pueda compartir?

Sí: ___ No: ____

Observaciones:

____
____
____
____

17. ¿Tiene contacto de alguna persona conocida que haya trabajado en la Planta de Procesamiento de Asbesto y que nos pueda dar el nombre y número de teléfono?

Nombre y Apellidos: ________________

Número de teléfono: ________________

Supplementary Materials 2: Review of Environmental Surveillance Records of the ACP. Procedure to access the complete files.

The search was conducted using the Environmental Management System (SAE, for its Spanish acronym) and the Public Documentary Information Management System (SIDCAR, for its Spanish acronym):

https://sae.car.gov.co/modulos/saepublico/Busqueda.aspx

In the SAE platform, by entering the facility's name in the "Username" field, which is one of the three available search parameters (file number, username, or user ID), the complete list of associated files within the platform is retrieved. No additional filters or keywords were necessary, as this query returned the complete set of records related to the facility.

The document types included environmental permits (some of which were incomplete) and administrative sanction case files. To obtain the complete records, incomplete information and key records that were not available in the system —such as the Unified Environmental Manufacturing Registry (RUA)— three formal right-to-petition requests were submitted to the CAR and to the National Institute of Hydrology, Meteorology, and Environmental Studies of Colombia (IDEAM) in accordance with Article 23 of the Colombian Constitution. All petitions received official responses.

Once the CAR petitions were answered, complete files were downloaded using the CAR official response number in the SIDCAR platform:

https://sidcar.car.gov.co/Modulos/Buzon/ConsultaPublica.aspx

Each right-to-petition with their specific answers and each number of environmental files are listed in table S2.1 and S2.2. However, as the information is public, the response can be consulted using the CAR and IDEAM official answer numbers that are available in table S2.1.

All case files associated with the ACP that were available in the CAR databases at the time of the review were included. No files were excluded, and no duplicate case files were identified. Incomplete or unavailable information was retained as a limitation of the available records rather than excluded from the review. Each retrieved document was manually reviewed by two authors (MG and LB), who divided the records for initial screening and information extraction. MG supervised

the review and was consulted whenever clarification was required regarding the interpretation or classification of information, with decisions reached by consensus. The extracted information was subsequently consolidated and cross-validated by a third author (AR), who verified each extracted statement against the corresponding content in the original case files to ensure its accuracy and consistency.

All retrieved records were categorized according to the classification established under Colombian environmental law (Decree 1076 of 2015) and by the type of administrative process defined by the CAR: (i) drinking water supply, (ii) wastewater discharges, (iii) air quality, (iv) soil resources, and (v) forest resource utilization. Although these platforms provide public access to environmental information, consultation may be restricted from outside the country due to virtual private network (VPN) limitations.

Table S2.1. Right-to-petitions to the CAR and IDEAM institutions. The first petition listed retrieved the complete environmental records from the CAR authority.

**Right-to-Petition list**						
**No.**	**Entity**	**Source**	**Type of document**	**Resources/Topic**	**Petition**	**Response**
**1**	**CAR**	**Right-to-Petition request to CAR**	**Permit**	**Water**	**Access to the complete copy files available in the SAE platforms. Petition No: 20251098876**	**CAR provided digital copies of the requested files.**<br><br>**Formal Response: CAR No. 11252003704, received 11/09/2025.**
2.	CAR	Right-to-Petition request to CAR	Permit	Water	Access to the Wastewater Discharge Permit File No. 31848 (Volume 2), and Unified Environmental Manufacturing Registry (RUA) and Hazardous Waste Generators (RESPEL) Registration. Petition No: 20241064752	CAR provided digital copies of the requested files.<br><br>Formal Response: CAR No. 11242003020, received 02/08/2024.

Right-to-Petition list						
**No.**	**Entity**	**Source**	**Type of document**	**Resources/Topic**	**Petition**	**Response**
3.	CAR	Right-to-Petition request to CAR	Environmental Registry	Registry	Detailed information regarding the facility's waste management. Petition No.: 20251038468	CAR indicated that reports on hazardous waste generation within its jurisdiction for the period 2011–2022 are publicly available through the following link: https://www.car.gov.co/vercontenido/5339. These reports include consolidated information on generation, utilization, treatment, and/or final disposal of hazardous waste. The 2023 data are in the process of being published. However, the information is not available at the individual facility level.<br><br>Formal Response: CAR No. 20252020885, received 21/04/2025.
4.	IDEAM	Right-to-Petition request to IDEAM	Environmental Registry	Registry	First request: Digital copy of information uploaded to the system regarding the registration of hazardous waste generators by ACP. Petition No: 20259050052324	IDEAM stated that the information cannot be provided at an individual level, only as consolidated national data, according to Article 10, Disclosure of Information, which requires IDEAM to guarantee access to consolidated information on its website. Formal Response: IDEAM No. 20256040057591, received 11/04/2025.

Right-to-Petition list						
**No.**	**Entity**	**Source**	**Type of document**	**Resources/Topic**	**Petition**	**Response**
5.	IDEAM	Right-to-Petition request to IDEAM	Environmental Registry	Registry	Second request: Digital copy of information uploaded to the system regarding the registration of hazardous waste generators by ACP. Petition No: 20259050074114	IDEAM reiterated that the information cannot be disclosed because Article 18 (as amended by Article 1, Decree-Law 2199 of 2015) of Law 1712 of 2014 classifies individual data such as NIT, facility name, address, phone number, responsible person, login, and password as restricted, and therefore it cannot be supplied to third parties. Formal Response: IDEAM No 20256000073671, received 13/05/2025

Table S2.2. Environmental documents reviewed, classified by document type, topic and subject. For each Results section, the table lists the records used in the reconstruction, enabling traceability between the reported findings and their documentary sources.

CAR File No.	Entity	Type of document	Resources/ Topic	Total Volumes*	Total Pages	Subject	Results subsection		
							3.2.1. Wastewater management and discharges	3.2.2. Waste Management	3.2.3. Air emissions
31848	CAR	Permit	Water	4	672	Wastewater Discharge Permit			
623	CAR	Permit	Water	2	282	Surface Water Use Permit			
74817	CAR	Sanction	Soil	2	282	Impact on Soil Resource			
31850	CAR	Permit	Air	28	6080	Atmospheric Emissions Permit for Stationary Sources			
31849	CAR	Permit	Water	8	1527	Groundwater Exploration Permit			

CAR File No.	Entity	Type of document	Resources/ Topic	Total Volumes*	Total Pages	Subject	Results subsection		
							3.2.1. Wastewater management and discharges	3.2.2. Waste Management	3.2.3. Air emissions
53376	CAR	Permit	Water	3	531	Groundwater Exploration Permit	These files were part of the review but did not contain any relevant information to reconstruct potential asbestos releases to air, water and soil.		
62568	CAR	Permit	Water	1	144	Prospection and Groundwater Exploration Permit			
79197	CAR	Permit	Water	2	306	Groundwater Use Permit and Well Registration			
59077	CAR	Permit	Forest	1	55	Permit for the Use of Isolated Trees			
78383	CAR	Permit	Forest	1	93	Permit for the Use of Isolated Trees			
105671	CAR	Permit	Forest	1	90	Permit for the Use of Isolated Trees			

CAR File No.	Entity	Type of document	Resources/ Topic	Total Volumes*	Total Pages	Subject	Results subsection		
							3.2.1. Wastewater management and discharges	3.2.2. Waste Management	3.2.3. Air emissions
79461	CAR	Permit	Forest	1	78	Permit for the Use of Isolated Trees			
64734	CAR	Sanction	Air	1	152	Impact on Air Resource			
**Total pages reviewed:**					**10,292**				

*Each CAR case file may comprise one or more volumes. Total pages correspond to the cumulative number of pages across all volumes.

Supplementary Materials 3: Identification of areas of the facility with higher potential for asbestos exposures. General production flowcharts from raw material reception to the initial stage for each of the three production lines.

Table S3.1. Glossary for Fig 1 in the document, related to the General production flowchart from raw material reception to the initial stage of the three production lines. This figure was developed using information collected by the former workers that participated in the study.

**Process / Material**	**Notes / Definitions**
Transport by rail and heavy vehicles	Refers to the supply chain logistics for raw materials and finished products.
Manual unloading	Physical discharge of materials from transport vehicles, performed by workers.
Cement	Hydraulic binder used as the matrix in asbestos cement products.
Asbestos	Raw mineral fiber used in manufacturing
Water	For hydration of cement and fiber dispersion.
Silos	Vertical structures used for storing cement or other bulk powders.
Storage facilities	General storage of raw materials or intermediate products.
Overhead crane	Mechanical lifting system for handling heavy loads inside the facility.
Operator loading	Manual or semi-automatic loading of raw materials into the process line.
Laboratory mix design	Location for development of proportions and recipes for asbestos cement formulations.
Milling	Mechanical reduction of particle size of raw materials.
Asbestos bag shredding	Opening and disintegration of asbestos fiber bags prior to feeding in the process.
Weighing	Measurement of raw materials before mixing to ensure correct proportions.
Mixing	Homogenization of cement, asbestos, cellulose, and additives with water.
Cellulose	Organic fiber used to improve flexibility and dispersion in the mixture.
Hazemag mill	Brand/model of mill equipment used for raw material reduction.
Waste recovery	Utilization of process residues (offcuts, defective products) for recycling into the production line.

Process / Material	Notes / Definitions
Process offcuts	Residues generated during cutting, trimming, or shaping.
Storage tanks	Tanks used for intermediate storage of liquid suspensions or mixtures.
Solvents	Chemical agents used for cleaning or aiding dispersion.
Screening	Process of separating particles or fibers by size.
Felt bonding	Process of reinforcing asbestos-cement sheets with a felt substrate.
Format generation	Shaping of products into standardized dimensions (sheets, pipes, etc.).
Sheet manufacturing	Fabrication of flat asbestos-cement sheets.
Pipe manufacturing	Fabrication of asbestos-cement pipes using cylindrical molds.
Mold-based manufacturing	General process of shaping products using molds (various geometries).

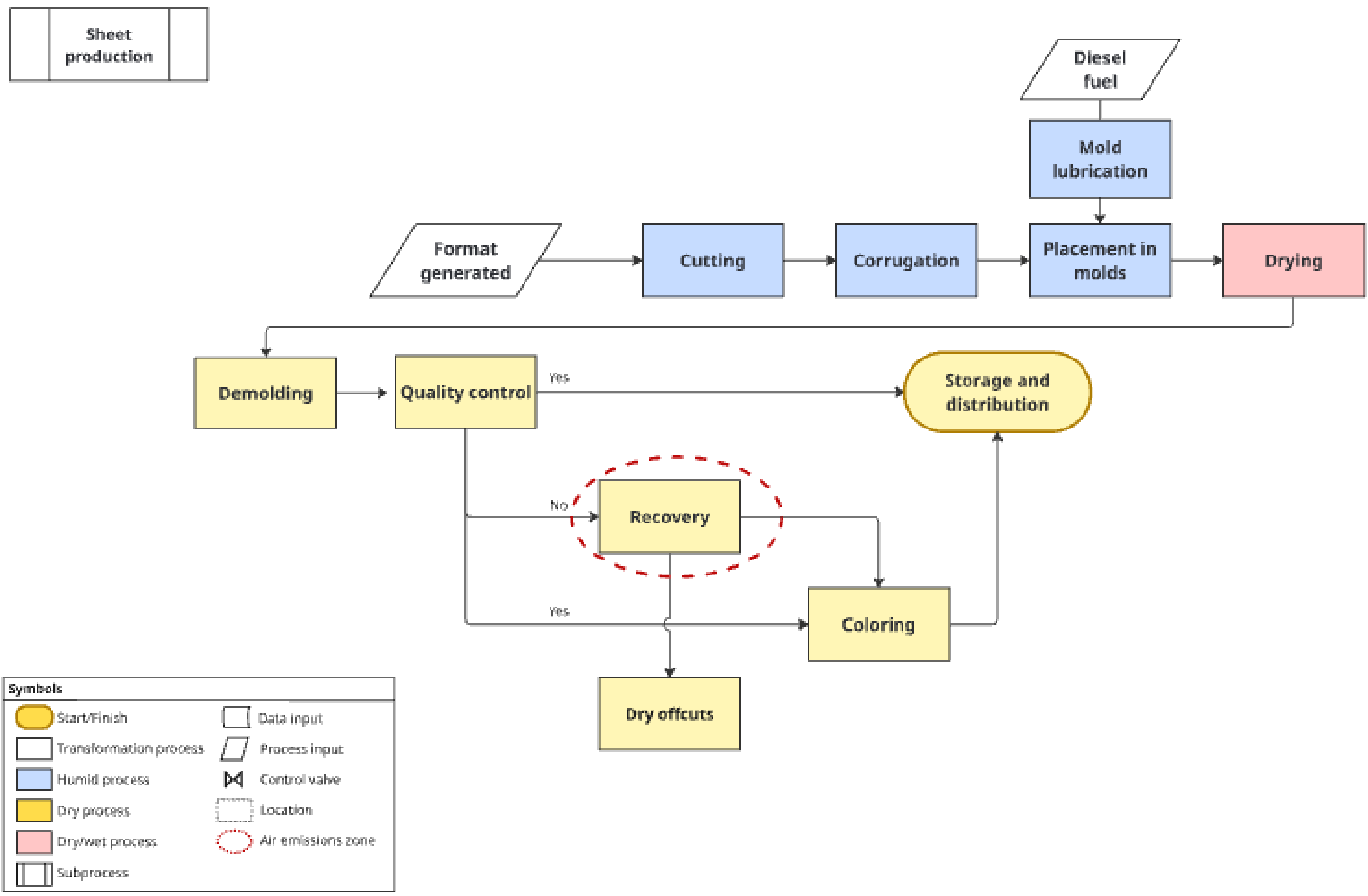

Fig. S3.1. Sheet production process once the format is generated until the finishing steps to get the final product.

Table S3.2. Glossary for Fig. S3.1, related to the sheet production process.

Process / Material	Notes / Definitions
Cutting	Process of dividing material into required dimensions.
Corrugation	Shaping process that produces waves or ridges, depending on the type of sheets.
Placement in molds	Positioning of the material within molds for shaping.
Diesel fuel	Refers to the addition of diesel fuel used for lubrication
Mold lubrication	Application of lubricants to facilitate demolding and prevent adhesion.
Drying	Removal of moisture content from the product.
Demolding	Extraction of the product from its mold once set.
Quality control	Inspection to ensure the product meets mechanical and dimensional standards.
Recovery	Process of reusing or recycling material or defective products.
Dry offcuts	Generation of solid waste or fragments generated during finishing.
Coloring	Addition of pigments or dyes to the material.
Storage and distribution	Logistics stage involving warehousing and delivery to end users.

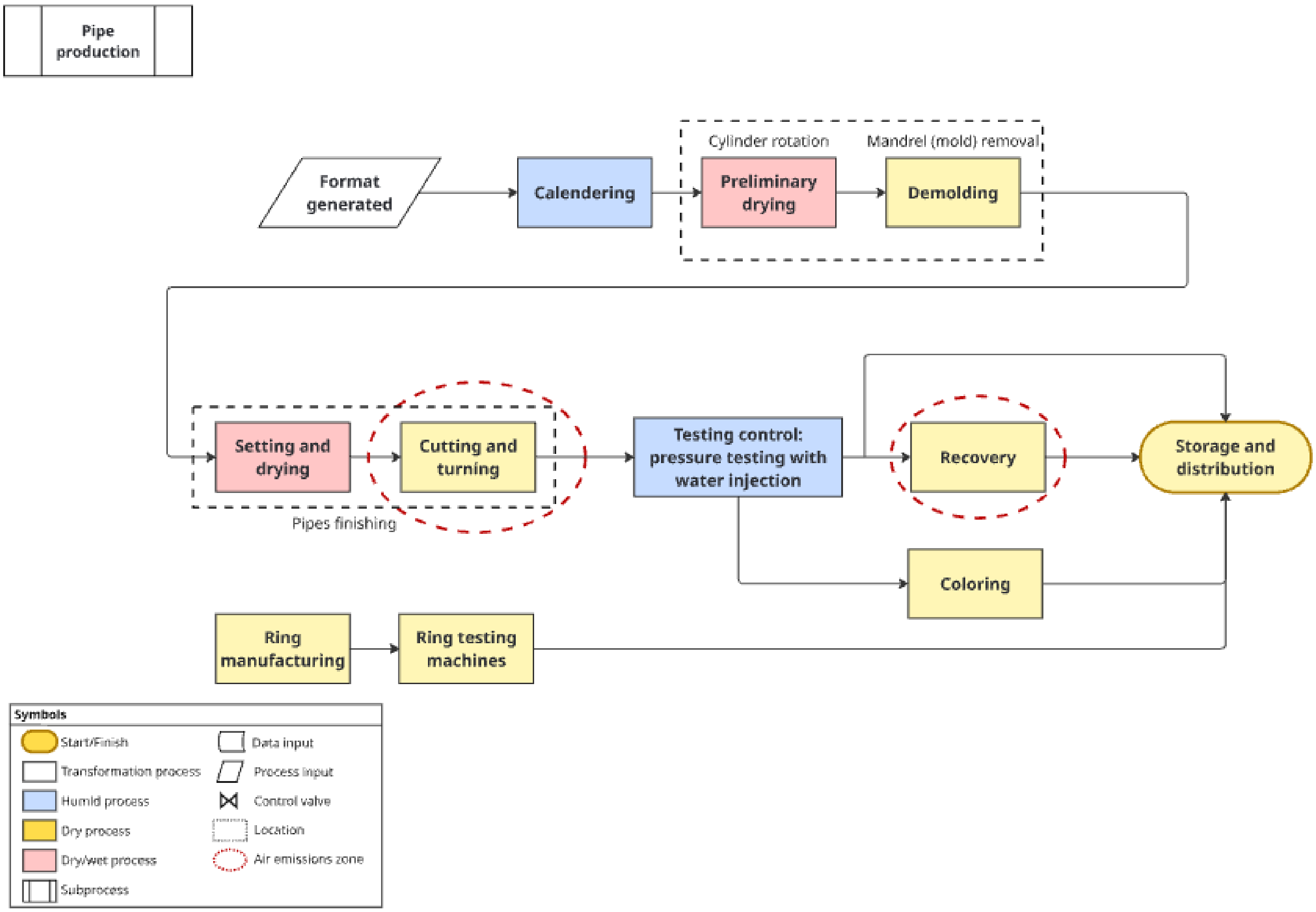

Fig. S3.2. Pipeline production process once the format is generated until the finishing steps to get the final product.

Table S3.3. Glossary for Fig. S3.2, related to <u>the pipeline production</u> process.

Process / Material	Notes / Definitions
Calendering	Process of passing material through rollers (calender) to control thickness and surface finish.
Preliminary drying	Initial drying stage before final curing.
Cylinder rotation	Rotation of the forming cylinder to shape the material.
Mandrel (mold) removal	Extraction of the mandrel (cylindrical mold used in pipe production).
Demolding	Extraction of the product from its mold once set.

Process / Material	Notes / Definitions
Placement in baskets and transfer by overhead crane	Handling and transport system for semi-finished products.
Setting and drying	Hardening of cementitious material followed by drying.
Cutting and turning	Machining processes to size and finish the product.
Testing control: pressure testing with water injection	Hydraulic test to ensure resistance and impermeability.
Recovery	Process of reusing or recycling material or defective products.
Coloring	Addition of pigments or dyes to the material.
Ring testing machines	Equipment used to evaluate the mechanical resistance of ring-shaped specimens.
Ring manufacturing	Specific production process for ring-shaped components.
Storage and distribution	Logistics stage involving warehousing and delivery to end users.

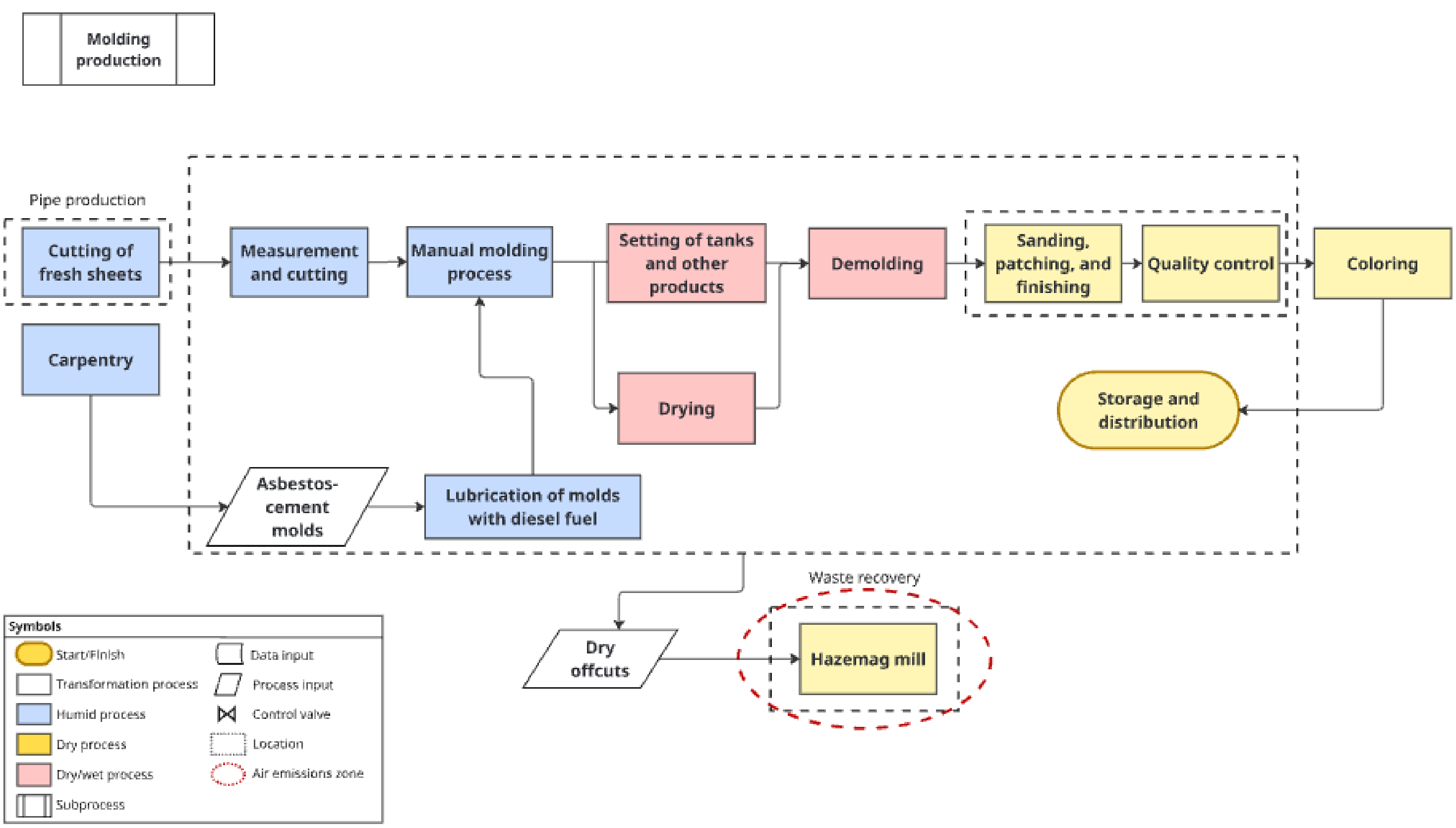

Fig. S3.3. Molding process once the format is generated until the finishing steps to get the final product.

Table S3.4. Glossary for Fig. S3.3, related to the molding process.

Process / Material	Notes / Definitions
Cutting of fresh sheets	Cutting performed while the asbestos-cement sheet is still in a plastic, unset state.
Measurement and cutting	Manual or mechanical sizing of products before or after curing.
Manual molding process	Traditional process in which operators shape products directly into molds.
Setting of tanks and other products	Refers to the curing/hardening of molded non-sheet products such as tanks or small structures (i.e., kennels).
Drying	Removal of moisture content from the product.
Demolding	Extraction of the product from its mold once set.
Sanding, patching, and finishing	Surface treatment and correction of defects to ensure product quality.
Quality control	Inspection of mechanical, dimensional, and visual properties.
Coloring	Addition of pigments or dyes to the material.
Carpentry	Fabrication and maintenance of auxiliary wooden components for molds and process supports.
Asbestos-cement molds	Specialized molds used to shape products from asbestos-cement mixtures.
Lubrication of molds with diesel fuel	Application of diesel oil to prevent adhesion during demolding.
Dry Offcuts	Residues or excess pieces removed during shaping.
Hazemag mill	Specific brand of milling equipment for raw material or waste processing.
Waste recovery	Utilization of process residues (offcuts, defective products) for recycling into the production line.
Storage and distribution	Logistics stage involving warehousing and delivery to end users.

Supplementary Materials 4: Reported quantities of asbestos waste (dust and fibers) sent for hazardous waste management and disposal.

Table S4. The table was compiled using information from the Unified Environmental Manufacturing Registry (RUA) file, a National Colombian Registry from CAR and IDEAM that was obtained through formal requests to the CAR. The classification type is done according to the Basel Convention on hazardous waste (i.e., classification in Annex I – Hazardous Wastes by Processes or Activities), adopted in Colombia under Decree 4741 of 2005. Reported only for years available in the registry; absence of a year does not imply zero generation.

**Residue type***	**Year**	**Max. Quantity (kg)**
Y36: Asbestos (dust and fibers)	2008	4,400
	2009	6,900
	2014	3,200
	2020	232
	2022	1,000
	2023	1,550

Supplementary Materials 5: Triangulation of former-worker testimonies and environmental regulatory records regarding asbestos-related practices and potential release pathways at the Sibaté asbestos-cement plant

No.	Practices	Reported by Former workers	Documented in Environmental regulatory records	Supported by both sources	Description
1	Production process following the Hatschek Process			X	Combining dry and wet stages. The production process is described in the environmental files and also described by former workers
2	Raw asbestos reception, unloaded and carried by former workers	X			Workers described manual unloading from rail wagons or trucks. Bags were manually unloaded from rail wagons until approximately 1980, and later from trucks. Workers carried asbestos sacks on their shoulders when mechanical systems were unavailable
3	Manual unloading and transport of asbestos sacks generated visible dust	X			Workers repeatedly described visible dust during sack handling
4	Opening asbestos bags was a potential point of fiber release	X			Workers identified bag opening as a key moment of fiber release
5	Milling of raw asbestos as one of the most dust-intensive tasks	X			Workers consistently identified unloading, storage, and milling as the most dust-intensive activities
6	Production included slurry preparation, dewatering, preform formation, sheet production, pipe production, and molding			X	Workers described the sequence and tasks in the three production lines that is consistent with the environmental files

No.	Practices	Reported by Former workers	Documented in Environmental regulatory records	Supported by both sources	Description
**7**	Finishing operations in pipe production were associated with higher airborne asbestos dust concentrations	**X**			Workers' accounts consistently described the pipeline production as an important source of asbestos fiber release
**8**	Dry sweeping was routinely used for cleaning	**X**			Workers reported routine cleaning practices such as dry sweeping. They reported that handling settled dust could resuspend asbestos fibers
**9**	Waste handling: manipulation of dry and friable material	**X**			This condition was described by workers as part of residue collection and recycling.
**10**	The types of asbestos used varied over time.	**X**			Five workers identified asbestos by mineral name; six identified it only by color; two did not know the type used.
**11**	Ventilation controls and information regarding personal protective equipment (PPE)	**X**			Local exhaust systems were introduced between the late 1970s and early 1980s. Information regarding engineering controls such as dust controls, and PPE are available only at plant-level information
**12**	Industrial and domestic wastewater were discharged outside the ACP.		**X**		CAR records documented long-term discharges of industrial and domestic wastewater.
**13**	Wastewater was discharged into the El Muña Reservoir and nearby water bodies.			**X**	CAR records documented discharges to the reservoir and nearby water bodies. The place was referred by former workers as "places to discharged or landfill" three times in the surveys.

No.	Practices	Reported by Former workers	Documented in Environmental regulatory records	Supported by both sources	Description
**14**	Location of the industrial wastewater pipeline of the ACP			X	Recorded coordinates identified the IWW pipeline south of the facility. Former workers identified in the maps the location of the industrial wastewater treatment plant in the south of the facility, close to El Muña.
**15**	Additional discharge points releases of untreated industrial and domestic wastewater		X		CAR identified these discharge points into a drainage channel connected to the Bogotá River (1989)
**16**	Industrial wastewater treatment with a complete recovery and recirculation system		X		First proposed in 1981, authorized operations in late 1988, with operational failures reported in 1989. CAR ordered cessation of the untreated discharges identified in 1989.
**17**	Untreated wastewater discharges persisting until the late 1990s.		X		CAR records indicated that untreated discharges continued despite the proposed treatment and recirculation system.
**18**	Environmental oversight was absent or undocumented before 1980.		X		No CAR documentation was available for the period before 1980, despite plant operation beginning decades earlier.
**19**	Sludge recovery of 85-90%. The remainder were disposed in Soacha landfills			X	The 85–90% sludge recovery estimate was documented only in the CAR records. However, both sources identified Soacha as a location where sludge and/or other residues from the facility were disposed of.
**20**	Intermittent wastewater discharges until 2011		X		CAR documented wastewater discharges for emergency cases. By 2017 no active discharges were reported.

No.	Practices	Reported by Former workers	Documented in Environmental regulatory records	Supported by both sources	Description
**21**	Sludge hazardous characterization and classification		**X**		CAR files stated that a Toxicity Characteristic Leaching Procedure (TCLP) was required to test sludge toxicity. By 2009 the company reported 100% sludge recirculation so the test was not necessary. No official classification of the sludge as hazardous waste was found.
**22**	Solid industrial waste to fill flood-prone or eroded areas		**X**		CAR files evidence this practice in 1982
**23**	Disposal of asbestos-containing waste outside the plant			**X**	Workers provided information on asbestos-landfill practices and possible disposal sites around Sibaté. The environmental surveillance records from CAR reported landfilling in Sibaté and nearby municipalities.
**24**	Additional potential disposal sites are described.			**X**	Behind the ACP, La Macarena Sector, Sibaté, Soacha are reported in the files. Former workers referred the surroundings of the facility, Sibaté, Soacha and El Muña, as "places to discharged or landfill" residues.
**25**	Asbestos presence in soil in the surroundings of the facility		**X**		In 2019, a CAR technical inspection documented asbestos including chrysotile, crocidolite, and amosite in a land adjacent to the ACP
**26**	Transportation and redistribution of asbestos-containing waste upon request of residents	**X**			Former workers accounts suggest that waste materials (i.e., broken pipes, sheets, and fragments) were redistributed beyond the facility to be used as construction fill.

No.	Practices	Reported by Former workers	Documented in Environmental regulatory records	Supported by both sources	Description
					Workers reported transportation frequencies of up to 5 truckloads per week.
**27**	Disposal of asbestos-containing waste at El Muña Reservoir and nearby neighborhoods			**X**	Former workers reported the disposal of asbestos-containing waste at the El Muña Reservoir and in nearby neighborhoods. This is consistent with CAR case-file records from 1982 and 2016-2019, which describe waste disposal in the same area.
**28**	No asbestos-specific air monitoring data or fiber-concentration were identified		**X**		The environmental-record review found no asbestos-specific monitoring. Only brief reports of having a concentration < 0.1 f/cc in some locations. No detailed information regarding the sampling methodology.
